# All Suture Bone Tunnel Docking Technique for Distal Biceps Tendon Repair

**DOI:** 10.1002/atn2.70184

**Published:** 2026-07-23

**Authors:** Dustin J. Kress, Amber Lopez, Nicolas Artz, Alvarho Guzman, James Lee, Jonathan Suh, Patrick McGahan, Ajith Malige, James L. Chen

**Affiliations:** ^1^ Advanced Orthopedics and Sports Medicine San Francisco California U.S.A.

## Abstract

Distal biceps tendon ruptures result in significant functional deficits, including 40% to 50% loss of supination strength if left untreated. Current hardware‐based fixation methods achieve excellent outcomes but are associated with higher costs, 20% to 25% complication rates, and technical complexity. The bone tunnel technique represents a cost‐effective alternative that eliminates hardware‐related complications while maintaining anatomic restoration. This technique utilizes a single 5 cm anterior incision with protection of the lateral antebrachial cutaneous nerve. A bone tunnel is created at the anatomic footprint using a spade‐tip drill, followed by 7 mm reaming, sized 1 mm smaller than the tendon diameter. The tendon is secured with #2 FiberLoop (Arthrex, Naples, FL) whip‐stitch sutures, seated into the tunnel, and fixed by passing sutures around the radial aspect of the radius shaft and through the tendon with square knots. Key advantages include elimination of expensive implants, no intraoperative imaging requirements, preservation of natural tendon‐bone healing, and universal accessibility. This technique provides reliable fixation through biological integration with reduced complexity and cost compared with hardware‐based methods.

## Graphical abstract

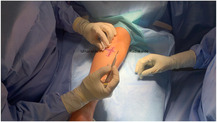

**VIDEO 1:** This video shows an all‐suture bone tunnel docking technique for distal biceps tendon repair. The patient is positioned supine, with the arm on a hand table and the elbow extended. A longitudinal incision is made over the radial tuberosity, with careful dissection to protect the lateral antebrachial cutaneous nerve. The distal biceps tendon stump is identified and secured with an Allis clamp. A #2 FiberLoop (Arthrex, Naples, FL) suture is used to whip‐stitch the tendon distally for manipulation. The radial tuberosity is exposed and prepared for bone tunnel creation. A spade‐tip drill is used perpendicular to the cortical surface to establish the tunnel entrance, followed by sequential reaming with a 7 mm over‐the‐top reamer. The FiberLoop suture is divided into 2 separate limbs and passed through the bone tunnel. The tendon is seated into the tunnel entrance, and the suture ends are passed around the radial aspect of the radius shaft and through the tendon itself using a free needle technique. Square knots are tied to secure the repair with appropriate tension. The repair is tested for stability through intraoperative range‐of motion‐testing, and layered wound closure is performed. Video content can be viewed at https://doi.org/10.1002/atn2.70184.

Distal biceps tendon ruptures are uncommon injuries affecting approximately 1.2 to 2.55 per 100,000 patient‐years, occurring predominantly in middle‐aged men during eccentric loading activities.^1^ When left untreated, complete ruptures result in substantial functional deficits, including 40% to 50% loss of supination strength and 20% to 30% reduction in flexion strength, making surgical repair the standard of care for healthy, active patients.^2^, ^3^

Current surgical approaches include single‐incision and dual‐incision techniques with various fixation methods, including cortical buttons, suture anchors, and interference screws.^4^ While these methods achieve excellent functional outcomes, they are associated with significant limitations: complication rates of 20% to 25%, high implant costs, and technical complexity requiring specialized equipment.^5^, ^6^, ^7^ Advanced fixation devices may cost several hundred dollars per case and are not universally available, particularly in resource‐limited settings.

The purpose of this Technical Note is to describe a bone tunnel technique for distal biceps tendon repair that eliminates hardware‐related complications while providing anatomic restoration at reduced cost and complexity compared with contemporary fixation methods.

## SURGICAL TECHNIQUE

### Patient Setup

The patient is positioned supine with the affected upper extremity placed on a padded hand table. A pneumatic tourniquet is applied to the proximal arm, and the extremity is prepared and draped in standard sterile fashion. The procedure is performed under general anesthesia.

### Surgical Approach

**FIGURE 1:**
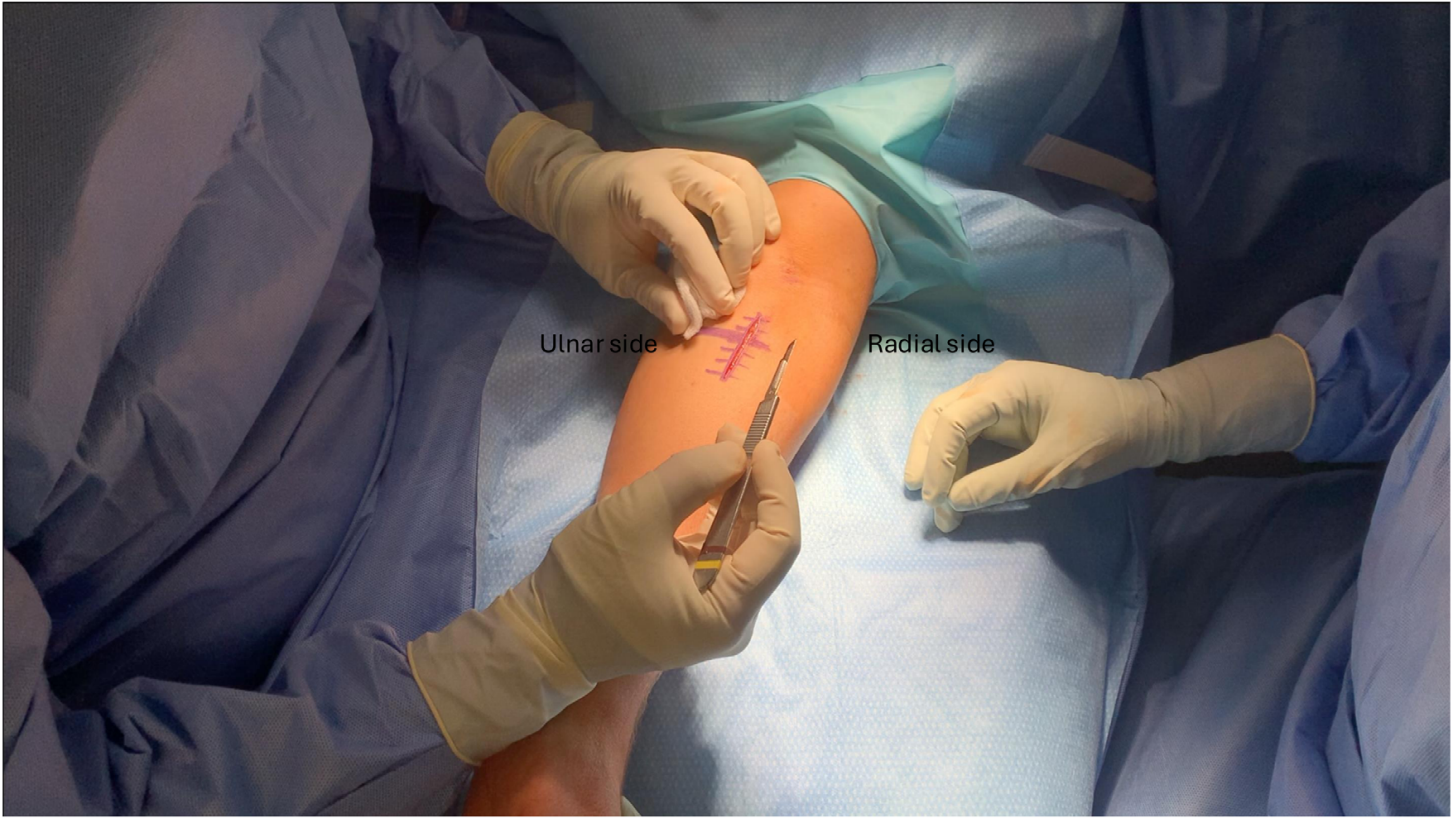
Intraoperative photograph showing surgical planning and patient positioning for left distal biceps tendon repair. The patient is positioned supine with the left upper extremity placed on a padded hand table. The forearm is maintained in supination to optimize exposure of the radial tuberosity and facilitate accurate anatomic tunnel placement. A 5 cm longitudinal incision is marked directly over the radial tuberosity using palpable anatomic landmarks. The incision will be made with sharp dissection through subcutaneous tissues, using standard technique. Careful identification and protection of the lateral antebrachial cutaneous nerve are critical during the surgical approach through this incision. The longitudinal orientation provides adequate exposure for tendon preparation, bone tunnel creation, and secure fixation while minimizing tissue disruption and optimizing cosmetic outcomes.

A longitudinal incision measuring 5 cm is made directly over the radial tuberosity (Video 1). Sharp dissection is carried through the subcutaneous tissues with careful identification and protection of the lateral antebrachial cutaneous nerve, which is retracted laterally (Figure 1). Blunt dissection using Metzenbaum scissors exposes the radial tuberosity while minimizing risk to surrounding neurovascular structures.

### Tendon Preparation and Assessment

**FIGURE 2:**
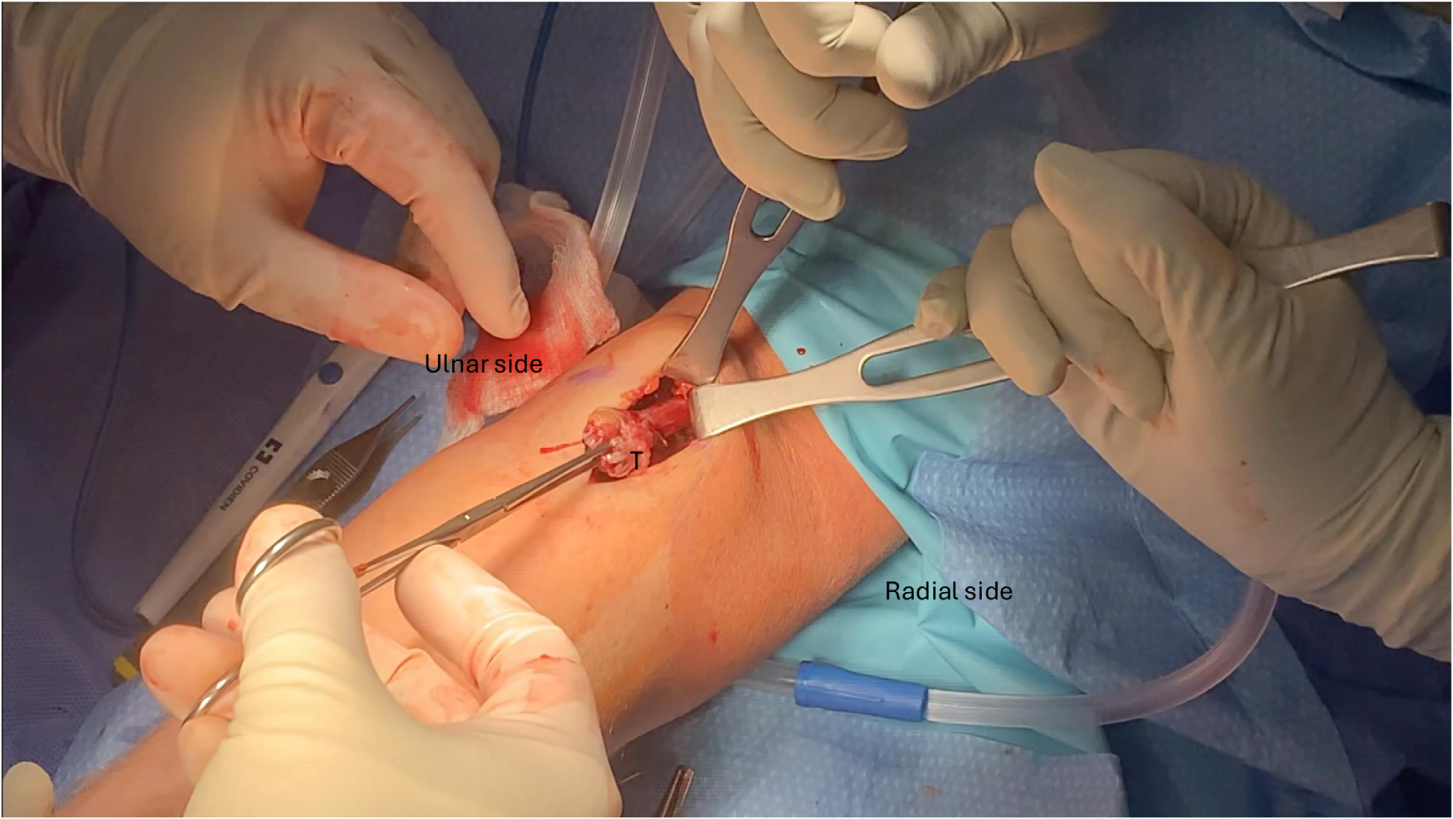
Intraoperative photograph showing identification of the ruptured distal biceps tendon during left distal biceps tendon repair. The patient is positioned supine with an anterior surgical approach. Following sharp dissection through subcutaneous tissues with careful identification and protection of the lateral antebrachial cutaneous nerve, the ruptured distal biceps tendon (T) has been identified and secured with an Allis clamp to facilitate manipulation and assessment. The tendon shows complete rupture from the radial tuberosity with proximally retraction. Retractors provide adequate exposure of the surgical field through the 5 cm longitudinal incision. The radial tuberosity lies deep in the wound and will be exposed for bone tunnel creation. Tendon quality assessment at this stage confirms adequate tissue for whip‐stitch preparation and anatomic repair.

**FIGURE 3:**
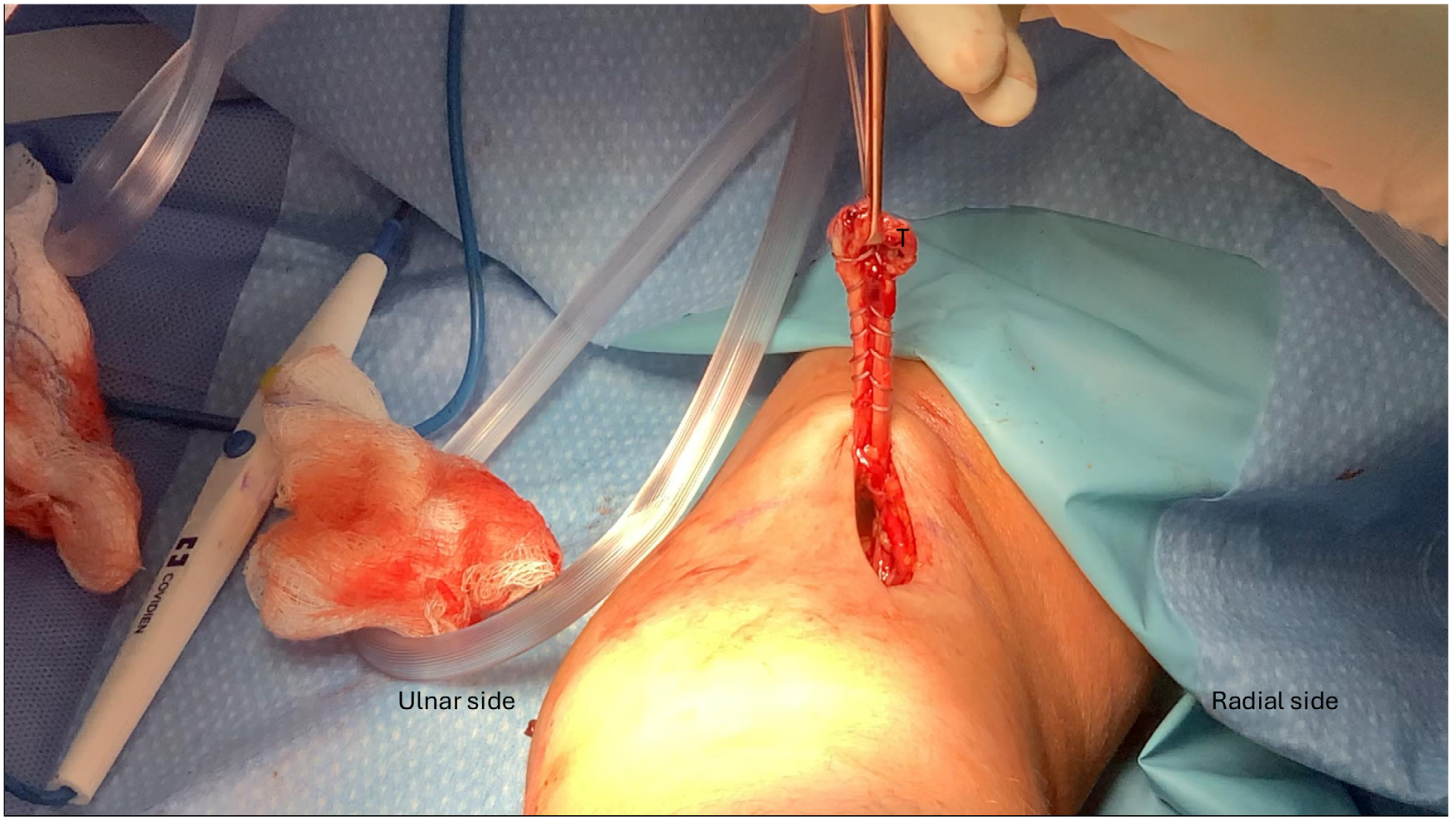
Intraoperative photograph showing tendon preparation with whip‐stitch technique during left distal biceps tendon repair. The patient is positioned supine with an anterior surgical approach. The ruptured distal biceps tendon (T) has been secured using a whip‐stitch pattern with #2 FiberLoop (Arthrex, Naples, FL) suture material to facilitate manipulation and ensure secure fixation. The whip‐stitch pattern is placed distally in the tendon end, creating multiple points of suture‐tendon interface to prevent suture pullout during tensioning. The intact suture loop is visible at the proximal aspect and will be divided to create 2 separate limbs for independent passage through the bone tunnel. The biceps muscle belly is visible proximally. The Allis clamp maintains tension on the suture during placement. Tendon diameter is measured at this stage (typically 7‐9 mm) to ensure appropriate bone tunnel sizing, which will be 1 mm smaller than the measured tendon diameter to optimize tendon‐to‐bone contact during fixation.

**FIGURE 4:**
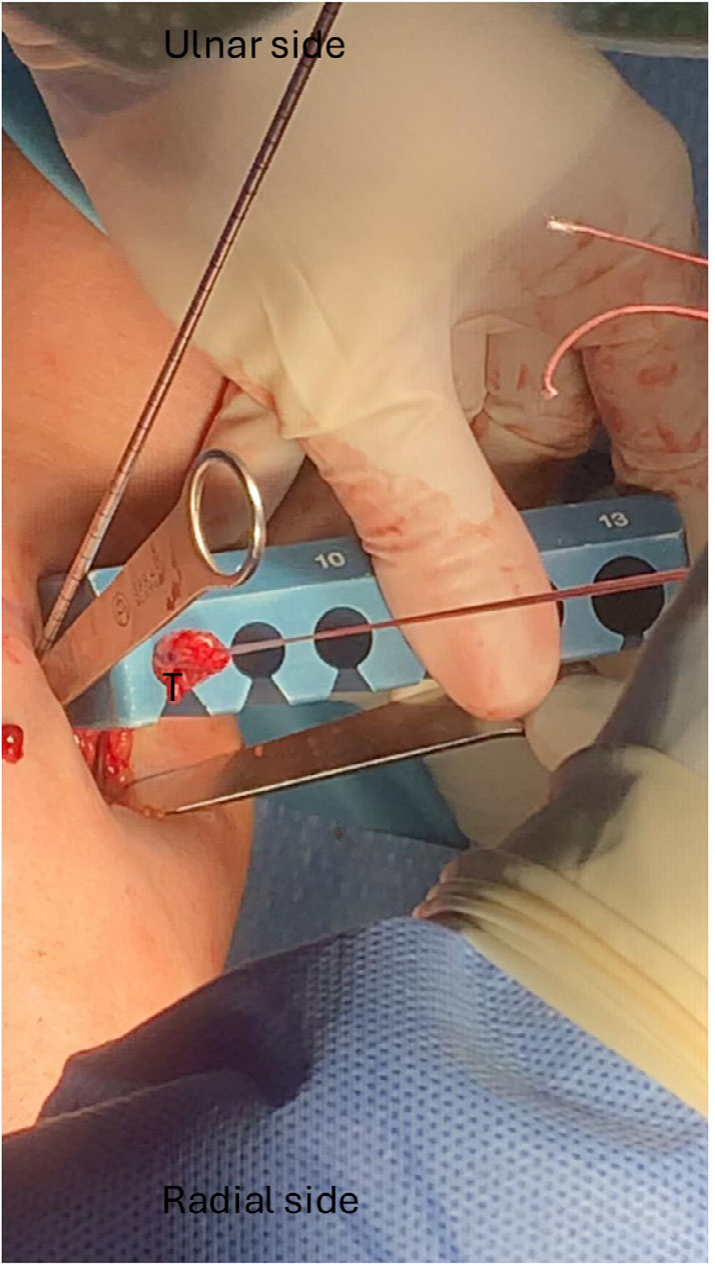
Intraoperative photograph showing tendon diameter measurement following whip‐stitch preparation during left distal biceps tendon repair. The patient is positioned supine with an anterior surgical approach. The whip‐stitched biceps tendon (T) is measured using a sizing gauge (SG), showing a diameter of 8 mm as indicated on the gauge markings. This measurement is performed after whip‐stitch placement and before bone tunnel creation to determine the appropriate tunnel size. Per the technique protocol, the bone tunnel will be reamed to 7 mm, 1 mm smaller than the tendon diameter, to ensure optimal tendon‐to‐bone contact and achieve secure press‐fit fixation without excessive compression. The #2 FiberLoop (Arthrex, Naples, FL) sutures are visible in the whip‐stitch pattern. Accurate tendon diameter measurement is critical for proper tunnel sizing and successful repair outcomes.

The ruptured distal biceps tendon is identified and assessed for tissue quality (Figure 2). The tendon end is secured using a whip‐stitch pattern with #2 FiberLoop (Arthrex, Naples, FL) suture material to facilitate manipulation (Figure 3). Tendon diameter is measured to ensure appropriate tunnel sizing, typically ranging from 7 to 9 mm in most patients (Figure 4).

### Bone Tunnel Creation

**FIGURE 5:**
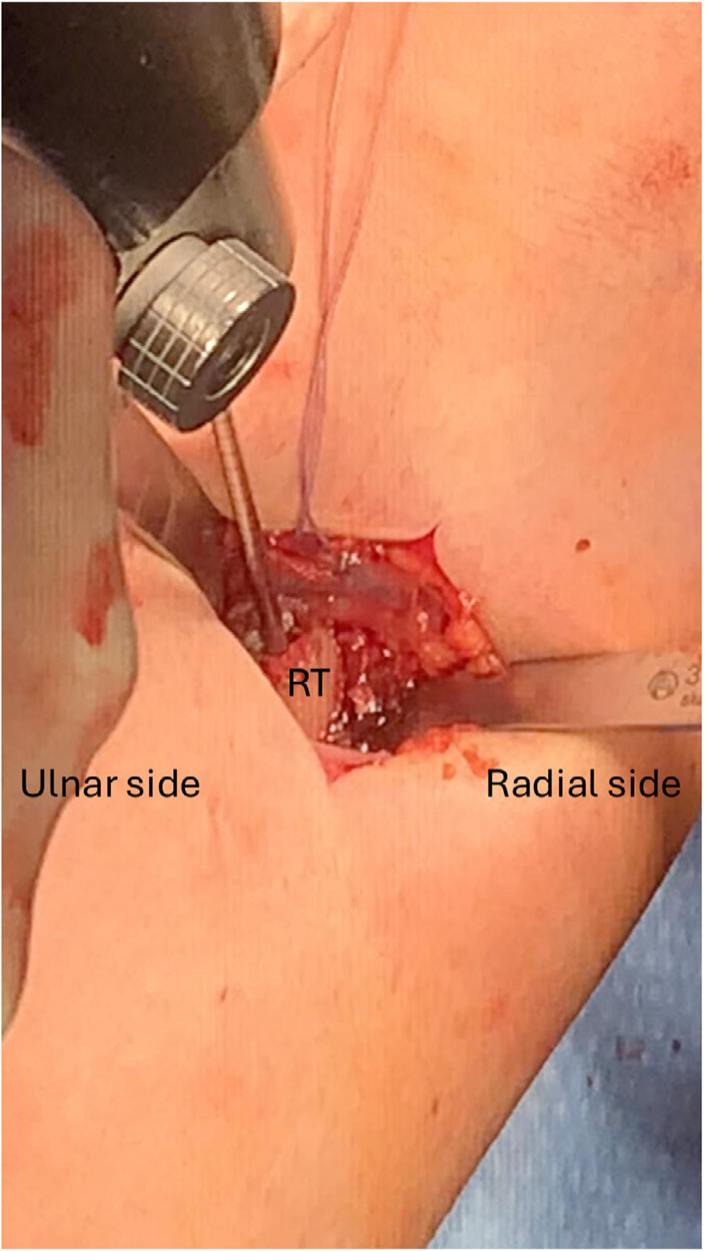
Intraoperative photograph showing bone tunnel creation at the anatomic footprint of the native biceps insertion during left distal biceps tendon repair. The patient is positioned supine, with the forearm maintained in supination to optimize exposure of the radial tuberosity. Following initial spade‐tip drill placement, sequential reaming is performed using a 7 mm over‐the‐top reamer placed perpendicular to the cortical surface of the radial tuberosity (RT). The reamer is advanced bicortically to create the complete bone tunnel through the radius. The tunnel is sized 1 mm smaller than the measured tendon diameter to ensure optimal tendon‐to‐bone contact and secure press‐fit fixation while preserving adequate bone stock. Sutures from the previously whip‐stitched biceps tendon are visible. Perpendicular drilling technique prevents tunnel enlargement and maintains parallel tunnel walls for optimal fixation strength and circumferential tendon contact.

**FIGURE 6:**
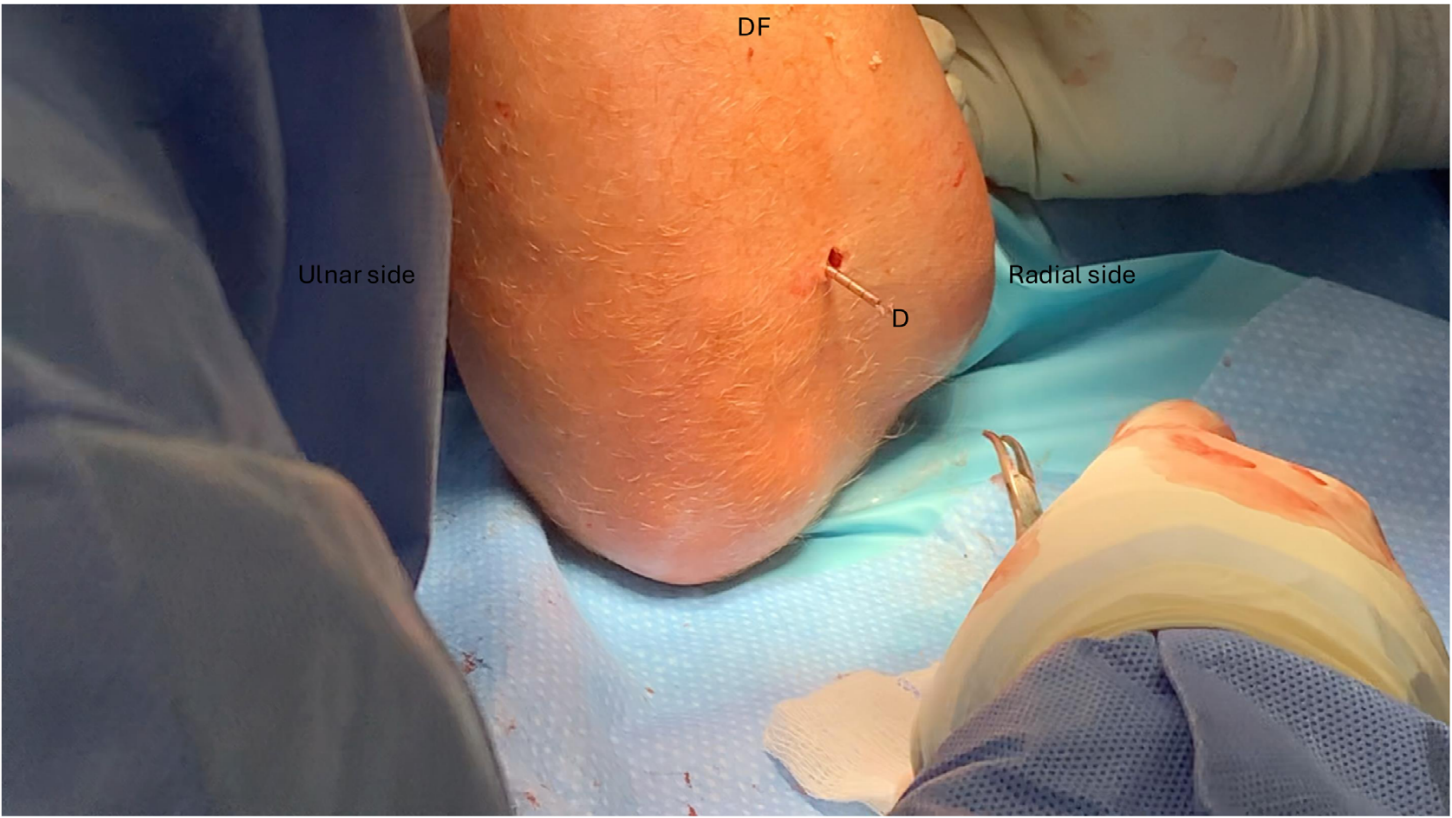
Intraoperative photograph showing verification of bicortical tunnel completion during left distal biceps tendon repair. Following anterior reaming with the 7‐mm over‐the‐top reamer, the spade‐tip drill bit (D) has been advanced completely through the radius and is visible exiting the posterior cortex on the dorsal aspect of the forearm (DF). The forearm has been pronated to visualize the dorsal exit point and confirm complete bicortical tunnel creation. This step verifies that the tunnel trajectory is appropriate and that the drill has successfully penetrated both cortices without complication. The bicortical tunnel allows for the passage of sutures and provides secure fixation. Visualization of the dorsal exit ensures the tunnel is properly oriented for the subsequent suture passage technique.

At the anatomic footprint of the native biceps insertion on the radial tuberosity, bone tunnel preparation begins with a spade‐tip drill bit placed perpendicular to the cortical surface to establish the tunnel entrance (Figure 5). The spade tip is advanced bicortically. After the proximal cortex is reamed, the spade tip Beath pin is advanced, shuttling a passing suture. Sequential reaming is performed using a 7 mm over‐the‐top reamer, sized 1 mm smaller than the measured tendon diameter, to create final tunnel dimensions while preserving adequate bone stock (Figure 6).

### Securing and Tensioning the Distal Biceps Tendon

**FIGURE 7:**
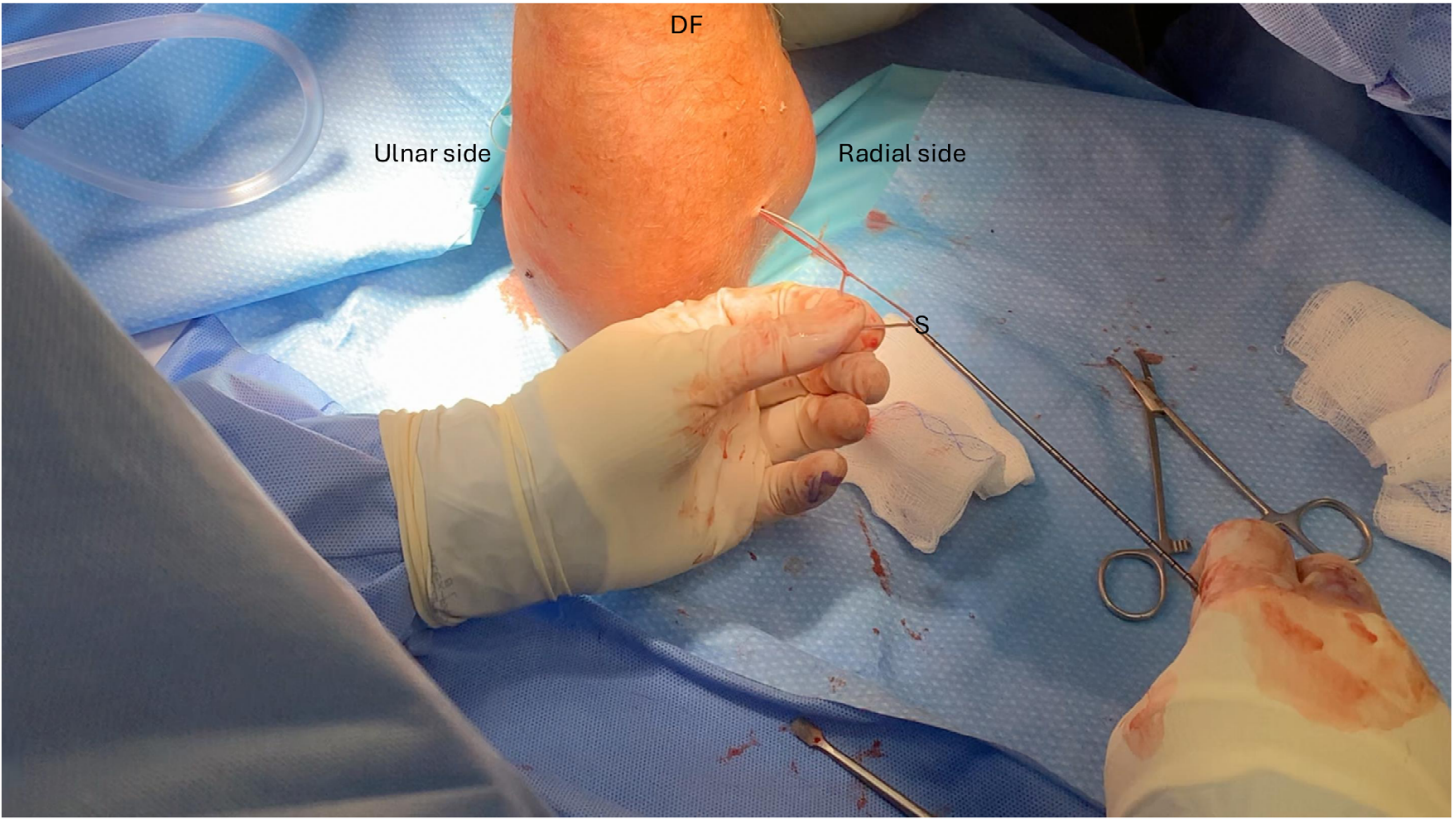
Intraoperative photograph showing successful suture passage through the bicortical bone tunnel during left distal biceps tendon repair. Following tunnel creation and tendon measurement, the previously placed #2 FiberLoop (Arthrex, Naples, FL) suture is divided to create 2 separate limbs for independent manipulation. Both suture limbs (S) have been passed through the bone tunnel from the anterior radial tuberosity through the posterior cortex and are visible exiting the dorsal aspect of the forearm (DF). The forearm is maintained in pronation to visualize and retrieve the sutures from the dorsal exit point. This step confirms successful tunnel passage and prepares the sutures for the subsequent wrapping technique around the radial aspect of the radius shaft. The ability to retrieve both suture limbs dorsally verifies that the bicortical tunnel is patent and appropriately positioned for the next fixation steps.

**FIGURE 8:**
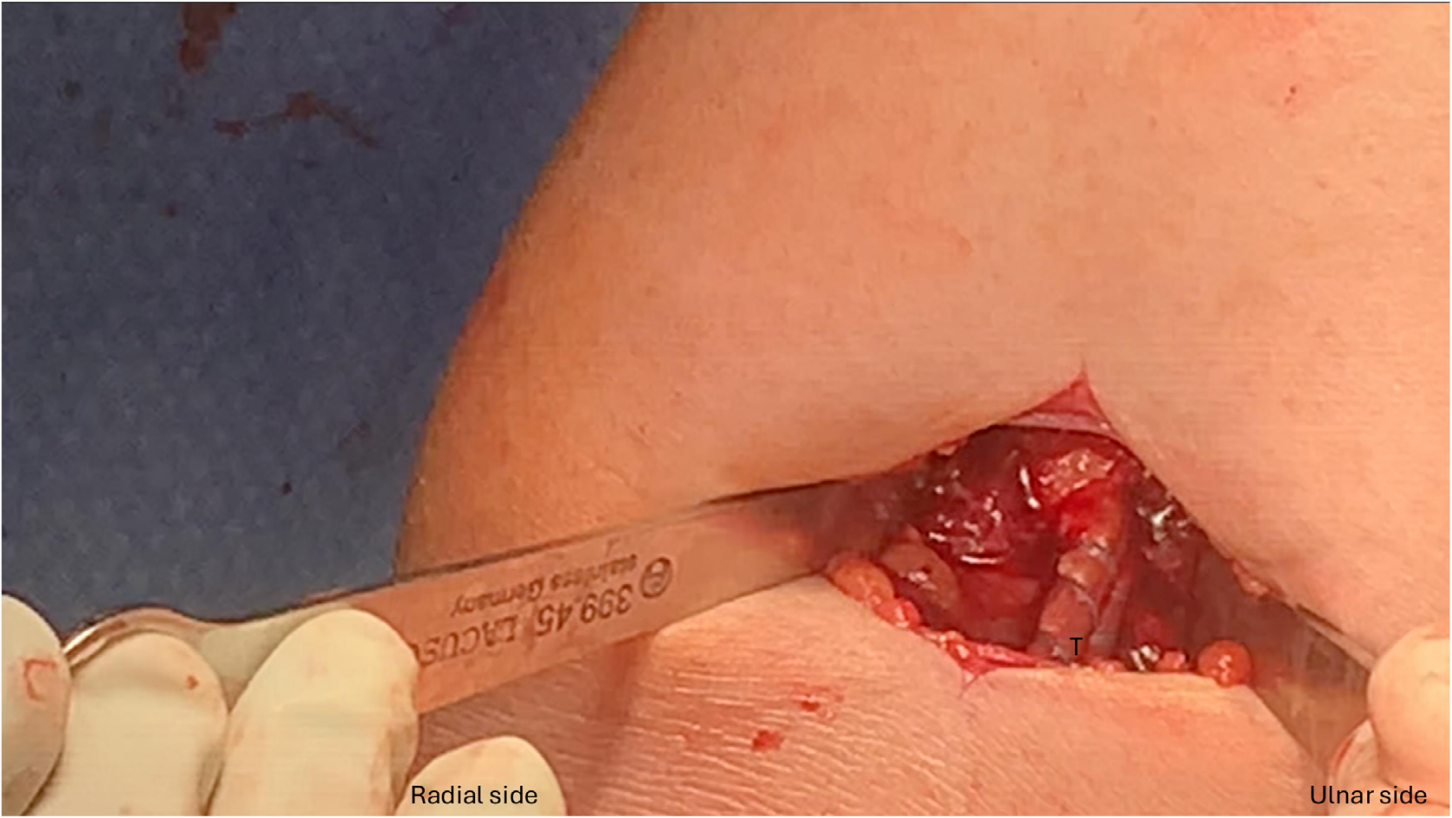
Intraoperative photograph showing tendon docking into the bone tunnel during left distal biceps tendon repair. The patient is positioned supine with the forearm maintained in supination. Following successful passage of the #2 FiberLoop (Arthrex, Naples, FL) sutures through the bicortical tunnel, tension is applied to the suture limbs from the dorsal aspect to pull the whip‐stitched biceps tendon (T) into the tunnel entrance at the radial tuberosity. The tendon is being seated into the anatomic footprint, with the whip‐stitch sutures visible. A retractor maintains exposure of the surgical field. The press‐fit technique ensures the 8 mm tendon is compressed into the 7 mm tunnel, creating optimal tendon‐to‐bone contact and primary stability before final suture fixation. This docking step restores the tendon to its native anatomic position at the radial tuberosity.

The previously placed #2 FiberLoop (Arthrex, Naples, FL) suture is divided to create 2 separate limbs for independent manipulation. Both suture limbs are passed through the prepared bone tunnel by the shuttling suture (Figure 7). The biceps tendon is then seated into the tunnel entrance, ensuring restoration of the anatomic insertion site and appropriate tendon‐to‐bone contact (Figure 8).

**FIGURE 9:**
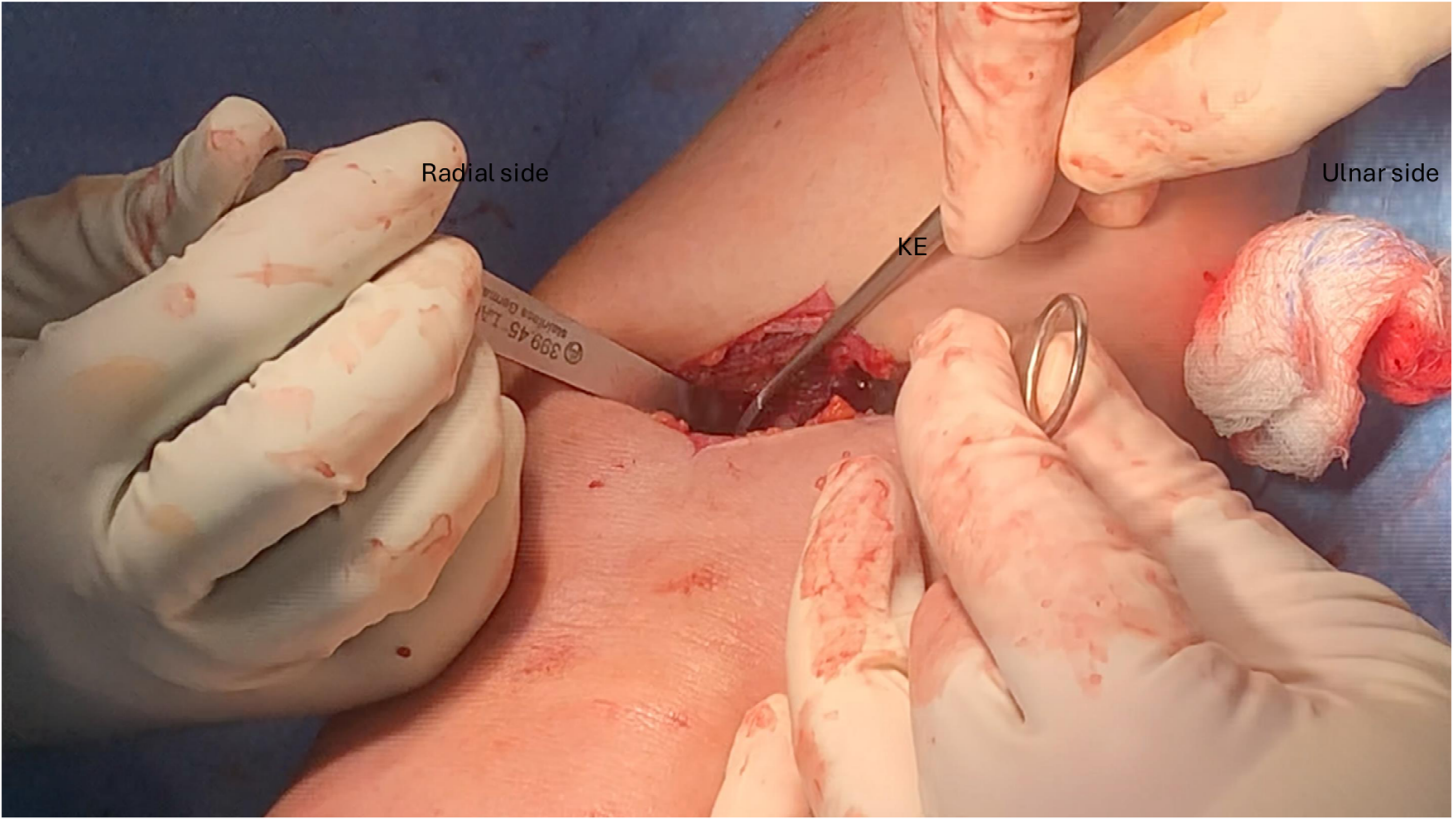
Intraoperative photograph showing preparation for suture passage around the radial aspect of the radius during left distal biceps tendon repair. The patient is positioned supine with anterior surgical approach. After the biceps tendon has been docked into the bone tunnel, preparation begins for passing the #2 FiberLoop (Arthrex, Naples, FL) suture limbs around the radial aspect of the radius shaft. A key elevator (KE) is used to bluntly create a safe subcutaneous passage around the radial side of the radius, protecting neurovascular structures and soft tissues while establishing the pathway for subsequent suture passage. This preparatory step is critical for the safe execution of the wrapping technique that distributes fixation forces around the radial aspect of the radius shaft. Following pathway creation, a right‐angle clamp will be used to pass the sutures through this prepared tissue plane.

**FIGURE 10:**
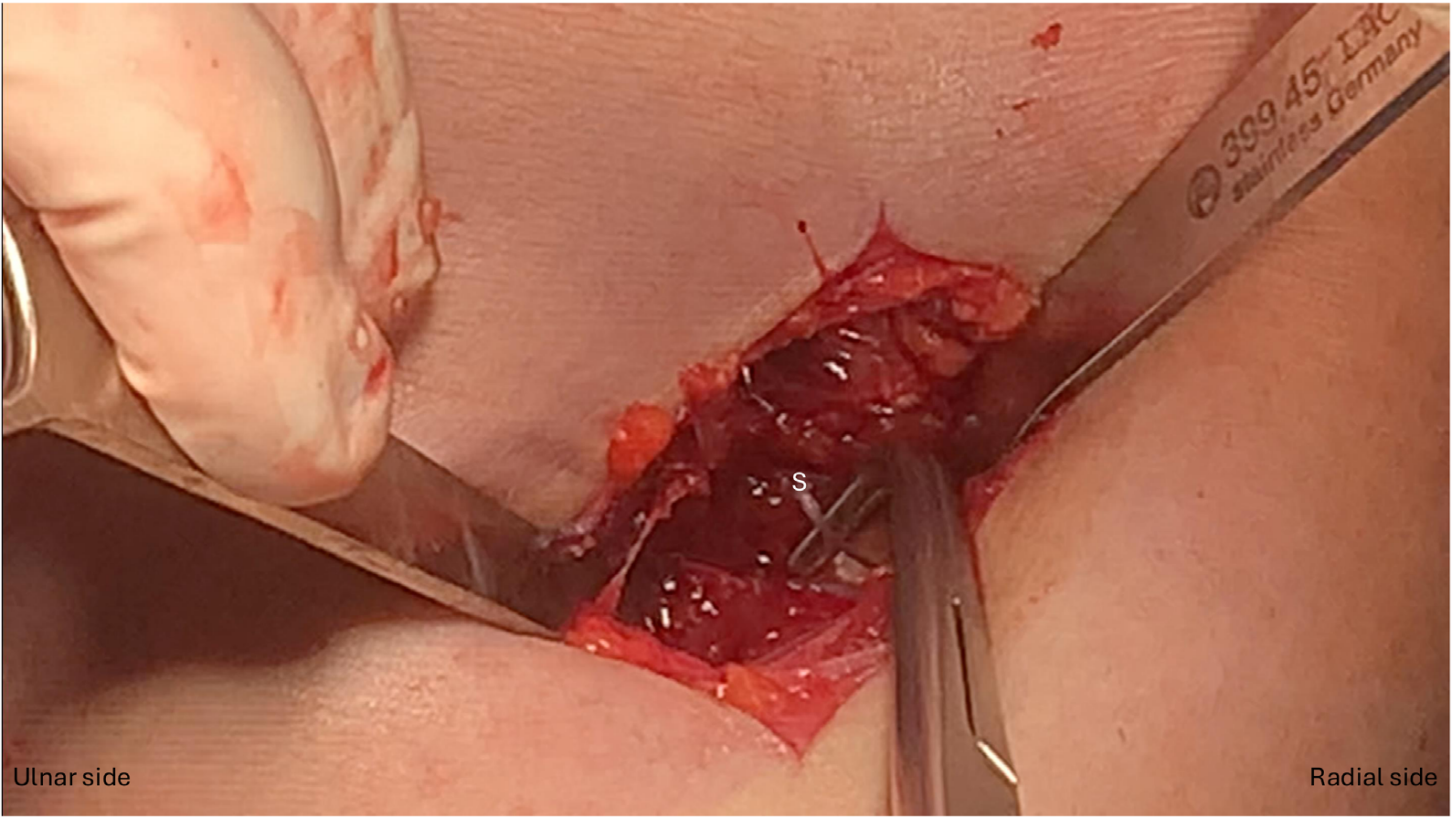
Intraoperative photograph showing active suture passage around the radial aspect of the radius during left distal biceps tendon repair. Following creation of a safe subcutaneous pathway with the key elevator, a right‐angle clamp is used to grasp and pull the #2 FiberLoop (Arthrex, Naples, FL) suture limbs (S) around the radial aspect of the radius shaft. The biceps tendon remains seated in the bone tunnel at the radial tuberosity. The right‐angle clamp facilitates controlled suture passage through the subcutaneous tissue plane while maintaining appropriate tissue protection. Both suture limbs are passed together around the radial side of the radius shaft and will return to the anterior incision site. This wrapping technique distributes fixation forces around the radial aspect of the radius rather than concentrating stress at a single point, which is a key technical feature of this all‐suture bone tunnel method.

**FIGURE 11:**
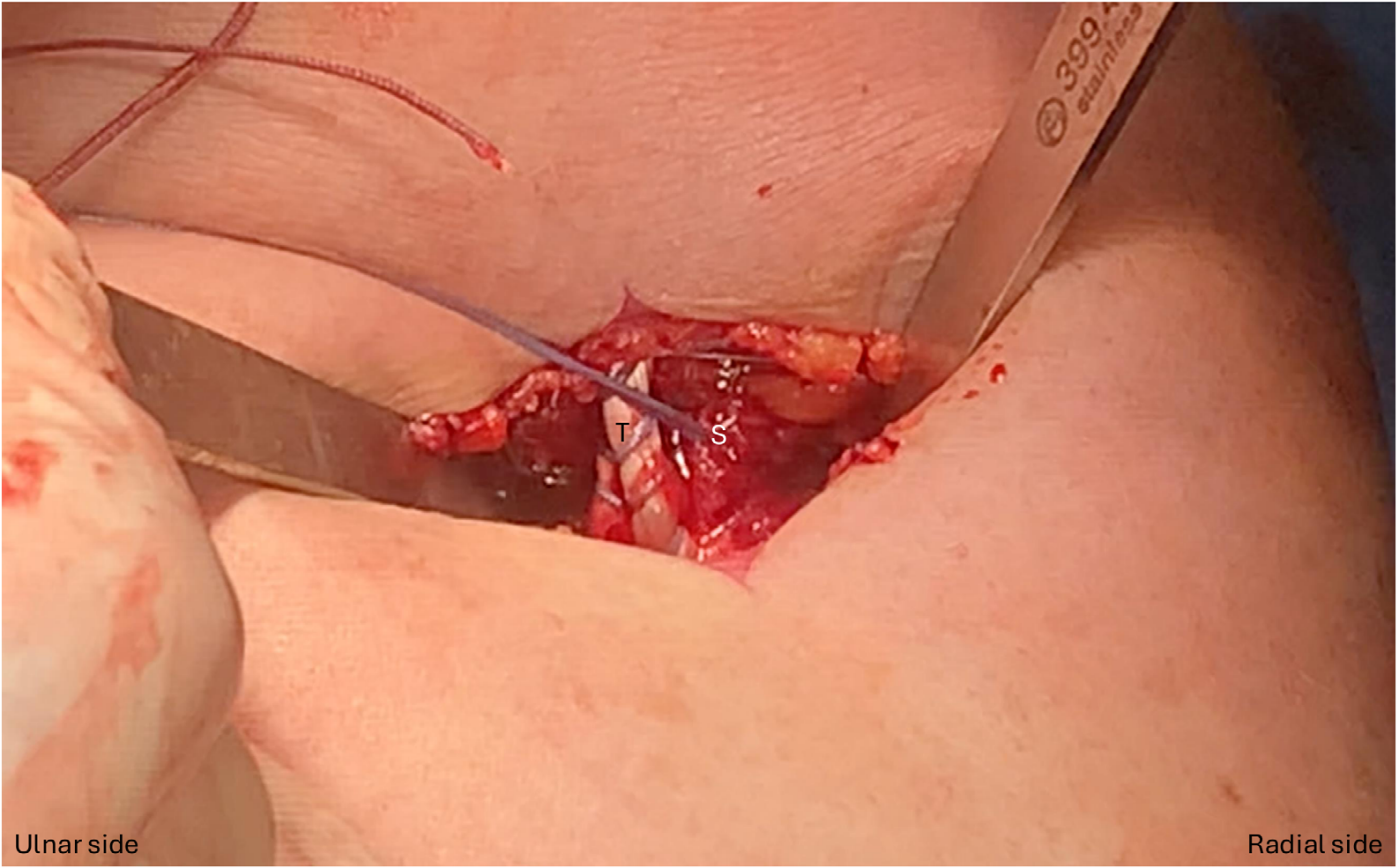
Intraoperative photograph showing suture positioning after passage around the radial aspect of the radius during left distal biceps tendon repair. Following the passage of both #2 FiberLoop (Arthrex, Naples, FL) suture limbs (S) around the radial aspect of the radius shaft, both limbs have returned to the anterior incision site. The biceps tendon (T) remains seated in the bone tunnel at the radial tuberosity. A clamp maintains tension on one suture limb while positioning for the next step. The wrapping around the radial aspect of the radius has been completed, distributing fixation forces around the radial side of the radius shaft. The suture limbs are now positioned for either passage through the biceps tendon itself using a free needle technique to create additional fixation points or for direct knot tying to secure the final repair.

A key elevator is used to bluntly create a safe subcutaneous passage around the radial side of the radius, protecting neurovascular structures (Figure 9). A right‐angle clamp is then used to pass both suture limbs through the prepared plane (Figure 10). This distributes fixation forces broadly rather than concentrating stress at a single point (Figure 11).

**FIGURE 12:**
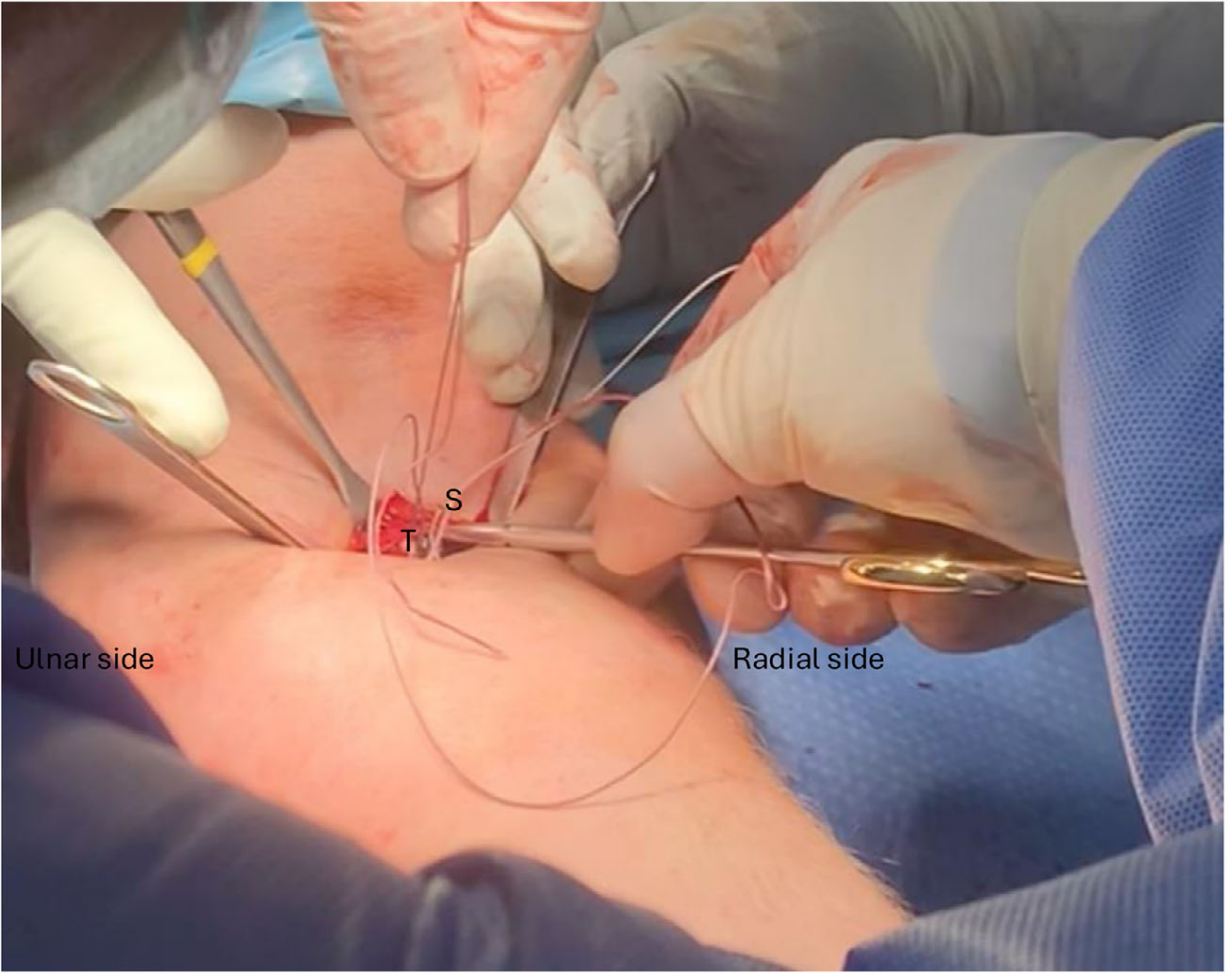
Intraoperative photograph showing the free needle technique for additional tendon fixation during left distal biceps tendon repair. Following the passage of both #2 FiberLoop (Arthrex, Naples, FL) suture limbs around the radial aspect of the radius shaft and their return to the anterior incision, the sutures are now passed through the biceps tendon (T) itself to create additional fixation points. A free needle loaded with one of the suture limbs (S) is being passed through the substance of the docked biceps tendon. This technique creates multiple points of suture‐tendon engagement beyond the initial whip‐stitch, enhancing overall repair security. Multiple passes are performed with each suture limb to optimize fixation strength. This step supplements the bone tunnel fixation and wrapping around the radial aspect of the radius by directly engaging the tendon substance, creating a robust multi‐point fixation construct. Following completion of the free needle passes, the suture limbs will be tied together with square knots to secure the final repair.

**FIGURE 13:**
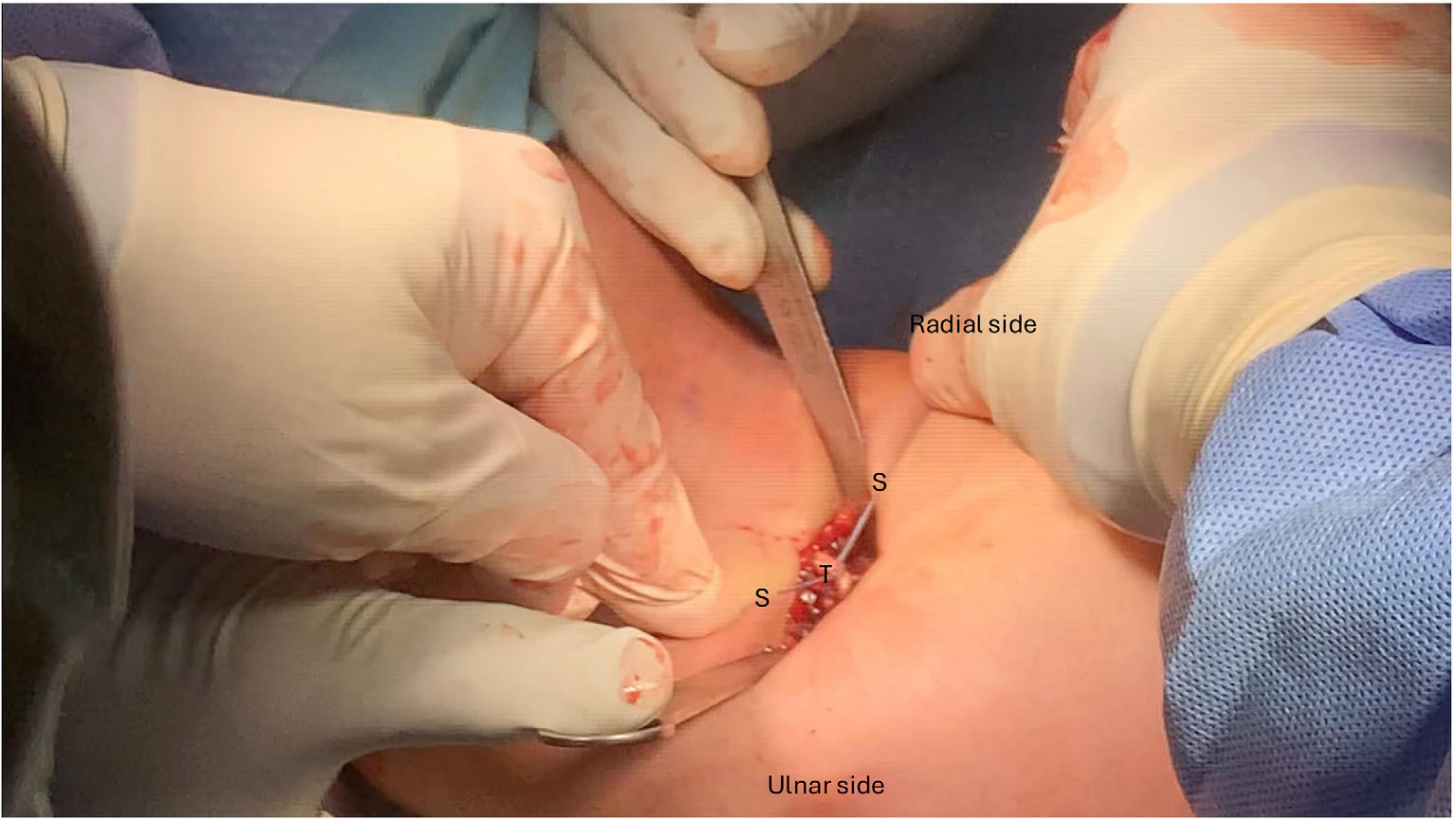
Intraoperative photograph showing knot tying to secure the final repair during left distal biceps tendon repair. Following passage of the #2 FiberLoop (Arthrex, Naples, FL) suture limbs (S) around the radial aspect of the radius shaft and through the biceps tendon (T) substance with the free needle technique, a series of square knots are tied to secure the repair with appropriate tension. The biceps tendon is visible seated in the bone tunnel. Multiple square knots are tied to ensure secure fixation, with careful attention to achieving appropriate tension that maintains the tendon firmly in the anatomic position without over‐compression. This final step completes the all‐suture bone tunnel construct, which achieves fixation through 3 mechanisms: press‐fit tendon‐to‐bone contact in the tunnel, distributed loading around the radial aspect of the radius shaft, and direct suture engagement of the tendon substance. Following completion of knot tying, intraoperative range‐of‐motion testing will be performed to verify repair integrity throughout elbow flexion and extension before wound closure.

Once the suture limbs are passed through the biceps tendon itself using a free needle to create additional fixation points (Figure 12). A series of square knots is tied to secure the repair with appropriate tension (Figure 13). Intraoperative range‐of‐motion testing confirms adequate repair strength without over‐constraint throughout elbow flexion and extension.

### Wound Closure and Postoperative Immobilization

Following copious irrigation and hemostasis, the wound is closed in layered fashion using 2 to 0 Vicryl for deep layers, 0 Vicryl for subcutaneous tissue, and 4 to 0 Monocryl for skin. A posterior splint is applied with the elbow at 90° of flexion. The arm is placed in a sling for additional support.

### Postoperative Care

For the first postoperative week, the patient remains in a posterior splint at 30° of flexion. Beginning with the second week, a removable hinged elbow brace locked at 90° is used, with passive range‐of‐motion exercises from 30° extension to 130° flexion performed 5 to 6 times daily. Active extension limits are progressively advanced from 20° at week 3 to 10° at week 4, with full extension permitted by week 5. The brace is discontinued at 6 weeks when light strengthening exercises begin with 1 kg weights. Progressive strengthening continues, with return to full activities by 3 to 6 months, depending on individual demands.

## DISCUSSION

Our experience with both bone tunnel and cortical button techniques shows that the bone tunnel method provides equivalent clinical outcomes while offering significant practical advantages. Cost represents the most obvious benefit‐implant costs account for approximately 29% of total surgical costs for distal biceps repair, with wide variability up to 121% of mean costs.^8^ The bone tunnel approach eliminates these implant expenses entirely while utilizing only basic suture materials. This economic advantage extends beyond implant costs to eliminate intraoperative fluoroscopy requirements, reducing operative time and radiation exposure.

Technical execution remains straightforward, as both techniques utilize identical surgical approaches and nerve protection strategies. Our comparative experience shows similar operative times to cortical button procedures, indicating that elimination of hardware placement simplifies rather than complicates the procedure. The key technical considerations are outlined in Table 1. Our modified fixation technique, incorporating circumferential suture passage around the radius and through the tendon, creates multiple points of fixation to enhance repair security.

**TABLE 1:** Technical Pearls and Common Pitfalls for Bone Tunnel Distal Biceps Tendon Repair. This Table Outlines Critical Technical Considerations to Ensure Successful Outcomes With the Bone Tunnel Technique

Technical Pearls	Common Pitfalls
Size tunnel 1 mm smaller than tendon diameter.	Oversized tunnel leads to poor fixation.
Use spade‐tip drill perpendicular to cortex.	Angled drilling creates weakened fixation.
Divide suture to create independent limbs.	Attempting to pass intact suture loop.
Pass sutures circumferentially around radius.	Inadequate suture wrapping reduces strength.
Use free needle for tendon passage.	Forcing sutures damages tendon tissue.
Test repair tension before final knots.	Skipping assessment leads to suboptimal repair.
Pass sutures circumferentially around radius.	Inadequate suture wrapping reduces strength.
Use free needle for tendon passage.	Forcing sutures damages tendon tissue.

The biological advantages center on preservation of the natural tendon‐bone healing interface. Unlike hardware methods that achieve fixation through cortical button engagement at a single point, the bone tunnel technique allows distributed healing throughout the tunnel length, providing multiple points of biological integration.^9^, ^10^ Long‐term studies show that bone tunnel fixation provides durable tendon‐to‐bone healing with excellent functional outcomes and low rerupture rates of approximately 2%.^5^, ^11^ This distributed healing pattern may contribute to the excellent long‐term durability observed while eliminating hardware‐related complications.

The bone tunnel technique for distal biceps tendon repair provides a reliable, cost‐effective alternative to hardware‐based fixation methods. By eliminating expensive implants while maintaining anatomic restoration, this approach addresses many limitations of contemporary techniques. When performed with attention to proper tunnel placement and tensioning, the bone tunnel repair consistently delivers satisfactory outcomes with low complication rates. This technique represents an excellent alternative for effective distal biceps repair with straightforward execution, offering surgeons an effective alternative to hardware‐based repairs at reduced cost and complexity.

Postoperative management is simplified without hardware‐specific restrictions. Our protocol allows early passive motion (30°‐130° flexion from week 1), reflecting confidence in repair strength. The absence of metallic implants eliminates magnetic resonance imaging artifacts, long‐term monitoring requirements, and revision complexity. Hardware removal, while uncommon, can be technically demanding and compromise bone stock, whereas bone tunnel repairs are easily revised if necessary.

Patient selection favors the bone tunnel technique when cost is important, fluoroscopy is unavailable, or patients prefer avoiding permanent implants. The technique applies equally to acute and chronic ruptures, although chronic cases may require additional mobilization. Contraindications are limited to poor bone quality or extensive bone loss that would compromise any fixation method. The clinical advantages and limitations of the bone tunnel technique are summarized in Table 2.

**TABLE 2:** Advantages and Disadvantages of the Bone Tunnel Technique for Distal Biceps Tendon Repair Compared With Hardware‐Based Fixation Methods

Advantages	Disadvantages
Cost‐effective ‐ eliminates expensive implants.	Repair dependent on suture integrity alone.
No intraoperative imaging required.	Requires meticulous surgical technique.
MRI compatible without artifacts.	Slightly higher risk of nerve neurapraxia.
Universal accessibility across settings.	May be challenging in poor bone quality.
Natural biological healing preserved.	Success depends on surgeon experience.

MRI, magnetic resonance imaging.

One potential disadvantage is the technique's dependence on suture integrity rather than mechanical hardware fixation. Additionally, single‐incision approaches may lead to slightly higher rates of lateral antebrachial cutaneous nerve neurapraxia compared with dual‐incision techniques, though this complication is typically transient.^12^ The technique also requires meticulous attention to tunnel placement and suture tensioning for optimal outcomes.

Although no single technique has shown clear superiority in the literature, our experience suggests the bone tunnel method achieves outcomes equivalent to hardware‐based repairs while providing practical advantages.^12^, ^13^ Future research should focus on long‐term comparative studies examining functional outcomes and cost‐effectiveness between fixation methods.

## DISCLOSURES

The authors (A.M., J.L.C.) declare the following financial interests/personal relationships which may be considered as potential competing interests: A.M. reports a relationship with Arthrex that includes: speaking and lecture fees. J.L.C. reports a relationship with Arthrex that includes: speaking and lecture fees. The other authors (D.J.K., A.L., N.A., A.G., J.L., J.S., P.M.) declare that they have no known competing financial interests or personal relationships that could have appeared to influence the work reported in this paper.
